# Obesity Treated by Sea-Water

**Published:** 1876-11

**Authors:** 


Obesity Treated by Sea-Water.—A writer in the *Paris Medical* recommends the treatment of obesity by the administration of sea-water, and the consequent residence by the seaside. The dose prescribed is a small glassful three times a day in a little fresh water or milk. Sea-baths are also recommended. We should think that the drinking and bathing might be advantageously combined to save time. In that way the dose, especially in surf-bathing, could be taken "plain," although the quantity could not, for obvious reasons, be regulated with exactness.—*Ibid.*

